# Assessing structural damage progression in psoriatic arthritis and its role as an outcome in research

**DOI:** 10.1186/s13075-020-2103-8

**Published:** 2020-02-03

**Authors:** Désirée van der Heijde, Dafna D. Gladman, Arthur Kavanaugh, Philip J. Mease

**Affiliations:** 1 Department of Rheumatology, Leiden University Medical Center, Albinusdreef 2, 2333 ZA Leiden, the Netherlands; 2 Krembil Research Institute, Toronto Western Hospital and University of Toronto, 60 Leonard Ave, Toronto, ON M5T 0S8 Canada; 3 Division of Rheumatology, Allergy, and Immunology, UC San Diego School of Medicine, 9500 Gilman Drive, La Jolla, CA 92093 USA; 4 Swedish Medical Center/Providence St. Joseph Health and University of Washington, 601 Broadway Suite 600, Seattle, WA 98122 USA

**Keywords:** Psoriatic arthritis, Structural damage, PsA radiographic scoring systems, Biologic agents, Peripheral arthritis

## Abstract

Psoriatic arthritis (PsA) is an immune-mediated, clinically heterogeneous disease characterized by arthritis, enthesitis, dactylitis, spondylitis, and psoriasis of the skin and nails. Persistent articular inflammation in patients with PsA can lead to structural damage, which can result in reduced physical function and quality of life. Structural damage can occur rapidly, and irreversible joint damage may be observed if patients are not treated promptly and appropriately. Therefore, evaluating therapeutic agents for their ability to inhibit structural progression has become increasingly important, with radiographic progression becoming a key efficacy outcome in clinical trials in PsA. Here, we review how structural damage and progression are assessed in clinical trials and the use of radiographic progression as a study outcome. We also discuss possible limitations in the current assessment of radiographic progression as well as areas of research that may improve the assessment of structural damage in clinical trials of PsA.

## Background

Psoriatic arthritis (PsA) is an inflammatory disease that develops in approximately 25% of patients with psoriasis [1] and is characterized by arthritis, enthesitis, dactylitis, spondylitis, and psoriasis of the skin and nails [2]. Persistent inflammation can damage patients’ cartilage and bone, leading to bony erosions and joint space narrowing or total joint destruction; in addition, some patients experience abnormal new bone growth [3, 4].

PsA was initially considered a mild disease, but evidence has shown that PsA has a substantial impact on patient quality of life and disability [5]. This is largely due to the structural damage associated with the disease, with a higher degree of joint damage correlating with greater disability and limitation of physical function [6]. Structural damage of joints is typically measured with conventional radiographs, and almost half of patients exhibit structural damage and functional impairment within 2 years of developing symptoms [7]. Many experience irreversible joint damage and disability as the disease progresses [7], and a diagnostic delay of > 6 months between symptom onset and the first rheumatologist visit has been linked to the development of peripheral joint erosions and worse long-term physical function [8]. In addition, radiographic damage has been found to be prognostic of shorter survival in patients with PsA [9].

Given the impact of joint damage on physical function, quality of life, and survival, inhibiting progression of structural damage is one of the main goals of therapy [10, 11]. Therefore, assessment of structural damage progression has become important in clinical trials evaluating treatments for PsA. Radiographic progression is currently the preferred outcome measure for structural damage progression, with therapeutic agents that inhibit radiographic progression considered to be disease modifying [10, 11]. This article reviews the various scoring systems used to assess radiographic damage in the peripheral joints of patients with PsA, as well as the use of structural damage as an outcome measure in clinical trials.

## Methods

Relevant articles were identified by conducting a targeted search of the medical literature available in PubMed. A broad search was initially performed using psoriatic arthritis in combination with various terms, including radiographic progression, scoring method, structural damage, radiography, and clinical trial. Identified articles were considered relevant if they presented data on the imaging and scoring of structural damage in psoriatic arthritis and the use of radiographic progression as an outcome measure in clinical trials. Additional articles were identified from the references cited in articles of interest and based on the authors’ knowledge of the published literature.

## Assessing structural damage in PsA

### Imaging techniques

Radiographs have been used to measure the extent of damage in clinical trials of PsA over the last 20 years and remain the standard approach for assessment of structural damage [3, 12]. Radiographs are used to determine the presence of periostitis (which has a “fluffy” characteristic in PsA), assess joint damage (e.g., bone erosions, osteolysis, subluxation, ankylosis), determine involvement of the sacroiliac joint and joints of the spine, and identify spurs at the entheses [3, 12].

Newer imaging modalities, including ultrasound, magnetic resonance imaging (MRI), and high-resolution micro-computed tomography, have recently been used to assess structural damage progression in PsA. However, these techniques are not frequently used to evaluate outcomes in clinical trials and will therefore not be discussed in this review.

### Current radiographic scoring methods for structural damage progression in PsA

Several semiquantitative scoring systems (Table 1) have been developed for the assessment of structural damage progression in PsA. Although they may be of limited use in the clinic, these scoring methods have proven very important in clinical research and have been used in many clinical trials to assess radiographic progression in the peripheral joints of patients with PsA (Table 2). These methods were originally developed for use in rheumatoid arthritis [3, 4] but have been modified for use in PsA [3], and some have been used in trials of biologic or targeted synthetic disease-modifying antirheumatic drugs [15, 17, 19–23, 25–27, 29, 30, 32]. The original scoring methods in rheumatoid arthritis involved assigning scores for bony erosions and joint space narrowing in various combinations of joints in the hands, wrists, and feet. Modifications to the scoring methods for use in PsA included adding joints involved more frequently in PsA and signs that are characteristic of PsA, such as the pencil-in-cup change, gross osteolysis, and ankylosis (Table 1) [3].

**Table 1:** Comparison of radiographic progression scoring methods in psoriatic arthritis trials

Scoring method	Total joints, *n* (range of score)	Locations (total joints, *n*)	Damage score per joint		Additional features recorded
Modified Steinbrocker [12, 13]	42 (0–168)	Hands (28) Wrists (2) Feet (12)	0 = normal 1 = soft tissue swelling/osteopenia 2 = erosion 3 = erosion plus JSN 4 = total joint destruction		–
Typical modified Sharp used for PsA [4]	Erosion, 54; JSN, 50 (erosion, 0–270; JSN, 0–200; total, 0–470)	Hands (28) Wrists (erosion, 14; JSN, 12) Feet (erosion, 12; JSN, 10)	Erosion: 0 = no erosion 1 = 1 discrete erosion or < 21% of joint 2 = 2 erosions or 21–40% of joint 3 = 3 erosions or 41–60% of joint 4 = 4 erosions or 61–80% of joint 5 = extensive destruction, > 80% of joint	JSN: 0 = normal joint 1 = asymmetrical/minimal narrowing 2 = definite narrowing with loss of ≤ 50% 3 = definite narrowing with loss of 51–99% 4 = absence of joint space, ankylosis	Pencil in cup; joint space widening
Sharp-van der Heijde for PsA [4]	52 (erosion, 0–320; JSN, 0–208; total, 0–528)	Hands (28) Wrists (12) Feet (12)	Erosion: 0 = no erosions 1 = discrete erosion 2 = large erosion not passing midline 3 = large erosion passing midline Scores can be combined if > 1 erosion per joint is present for a maximum score of 5 per joint in the hands and 10 per joint in the feet [Score of 5/10 if pencil-in-cup change or gross osteolysis present]	JSN: 0 = normal joint 1 = asymmetrical/minimal narrowing up to 25% 2 = definite narrowing with loss ≤ 50% 3 = definite narrowing with loss of 51–99% or subluxation 4 = absence of joint space, ankylosis, or complete luxation [Score of 4 if pencil-in-cup change or gross osteolysis present]	Pencil in cup; gross osteolysis
Psoriatic Arthritis Ratingen Score [14]	40 (destruction, 0–200; proliferation, 0–160; total, 0–360)	Hands (28) Wrists (2) Feet (10)	Destruction: 0 = normal 1 = ≥ 1 erosion, > 1 mm interruption of cortical plate, with up to 10% destruction of joint surface 2 = 11–25% destruction of joint surface 3 = 26–50% destruction of joint surface 4 = 51–75% destruction of joint surface 5 = > 75% joint surface destruction	Proliferation: 0 = normal 1 = bony proliferation of 1–2 mm or bone growth < 25% of original diameter 2 = bony proliferation 2–3 mm or bone growth of 25–50% 3 = bony proliferation > 3 mm or bone growth of > 50% 4 = bony ankylosis	–

*JSN* joint space narrowing, *PsA* psoriatic arthritis

**Table 2:** Assessment of radiographic progression in psoriatic arthritis trials

Study	Scoring method	Radiographic assessment time point, week	Mean change in radiographic score at week 24		Proportion of patients with no progression (mean change ≤ 0.5) at week 24, %	
			Placebo	Active treatment	Placebo	Active treatment
Etanercept vs placebo (phase 3) [15, 16]	Sharp	24 and 48	1.0^a^	− 0.03^a^	8.6^a,b^	33.8^a,b^
ADEPT (adalimumab vs placebo) [17, 18]	Sharp	24	1.0	− 0.2	71.1	91.0
IMPACT (infliximab vs placebo) [19]	SvdH	50	− 1.95 (PBO/infliximab)^c^	− 1.52^c^	84.8 (PBO/infliximab)^c^	83.8^c^
IMPACT 2 (infliximab vs placebo) [20]	SvdH	24 and 54	0.82	− 0.70	78.0	90.0
GO-REVEAL (golimumab vs placebo) [21]	SvdH	24 and 52	0.27	− 0.09	62.7^b^	77.7^b^
GO-VIBRANT (golimumab vs placebo) [22]	SvdH	24	2.0	− 0.4	43.0^b^	71.7^b^
RAPID-PsA (certolizumab pegol) [23, 24]	SvdH	12 and 24	0.28	0.06 (200 mg Q2W + 400 mg Q4W)	80.1	93.5 (200 mg Q2W); 90.4 (400 mg Q4W)
PSUMMIT-1 and -2 (ustekinumab vs placebo) [25]	SvdH	24 and 52	1.0	0.4	59.8^b^	66.5^b^
FUTURE 1 (secukinumab vs placebo) [26]	SvdH	16 or 24 and 52	0.57	0.08 (pooled secukinumab group)	75.7	82.3 (IV → 150 mg); 92.3 (IV → 75 mg)
FUTURE 5 (secukinumab vs placebo) [27]	SvdH	24	0.5	0.08 (300 mg + LD); 0.17 (150 mg + LD); − 0.09 (150 mg − LD)	73.6	88.0^.^(300 mg + LD); 79.8 (150 mg + LD); 83.8 (150 mg − LD)
SPIRIT-P1 (ixekizumab vs placebo vs adalimumab) [28]	SvdH	24 and 52	0.27 (PBO/IXEQ4W)^d^; 0.41 (PBO/IXEQ2W)^d^	0.54 (IXEQ4W/IXEQ4W)^d^; 0.09 (IXEQ2W/IXEQ2W)^d^; 0.32 (ADA/IXEQ4W)^d^; − 0.03 (ADA/IXEQ2W)^d^	94.1 (PBO/IXEQ4W)^d^; 65.7 (PBO/IXEQ2W)^d^	88.9 (IXEQ4W/IXEQ4W)^d^; 90.4 (IXEQ2W/IXEQ2W)^d^; 87.5 (ADA/IXEQ4W)^d^; 91.4 (ADA/IXEQ2W)^d^
ASTRAEA (abatacept vs placebo) [29]	SvdH	24 and 52	0.35	0.30	32.7^b^	42.7^b^
OPAL Broaden (tofacitinib vs adalimumab vs placebo) [30]	SvdH	12 months	Not reported by treatment arm. Range of change from baseline, − 0.07 to 0.09^a^		95.8 (PBO/TOFA 5 mg)^a^; 91.1 (PBO/TOFA 10 mg)^a^	95.9 (TOFA 5 mg)^a^; 94.9 (TOFA 10 mg)^a^; 97.9 (ADA)^a^
SEAM-PsA (methotrexate vs etanercept vs combination) [31]	SvdH	24 and 48	–	0.08 (MTX)^e^; − 0.04 (ETN)^e^; − 0.01 (MTX + ETN)^e^	–	89.4 (MTX)^b,e^; 94.7 (ETN)^b,e^; 94.7 (MTX + ETN)^b,e^

*ADA* adalimumab, *ETN* etanercept, *IXE* ixekizumab, *LD* loading dose, *MTX* methotrexate, *PBO* placebo, *PsA* psoriatic arthritis, *Q2W* every 2 weeks, *Q4W* every 4 weeks, *SvdH* modified Sharp-van der Heijde score for PsA, *TOFA* tofacitinib

^a^Mean change from baseline at 12 months

^b^No progression was defined as a mean change of ≤ 0 from baseline

^c^Mean change from baseline at week 50

^d^Mean change from baseline at week 52

^e^Mean change from baseline at week 48

#### Modified Steinbrocker global scoring method

The original Steinbrocker method classified patients with rheumatoid arthritis according to functional and radiographic damage and assigned each patient a score of 0 (normal) to 4 (total joint destruction) based on the patient’s worst joint [3, 12]. The University of Toronto Psoriatic Arthritis Clinic modified the method for use in PsA to include 42 joints in the hands, wrists, and feet and assigned each joint a single score from 0 to 4 to reflect the extent of both erosion and joint space narrowing, for a total score ranging from 0 to 168 (Table 1) [12, 13].

#### Sharp scoring method for PsA

The PsA-modified Sharp method typically assesses joints of the hands, wrists, and feet, including the second to fifth distal interphalangeal (DIP) joints of both hands (total score range, 0–470); a separate evaluation of erosions and joint space narrowing is also performed (Table 1) [3, 4]. Erosion is measured on a scale of 0 to 5 (total range, 0–270) and accounts for both the number of discrete erosions and percentage of joint involvement, with 0 defined as no erosion and 5 defined as extensive destruction involving > 80% of the joint. Extreme bone destruction (pencil-in-cup changes, gross osteolysis) may also be captured separately, but these are not included in the total erosion score. Joint space narrowing is scored on a scale of 0 to 4 (total range, 0–200), with a normal joint assigned a score of 0 and the absence of joint space or evidence of ankylosis a score of 4. Joint space widening is scored separately; however, it is not included in the total joint space narrowing score. Similarly, other PsA-associated structural damages (e.g., periostitis, tuft resorption) are recorded separately, but are also not included in the total score value.

#### Sharp-van der Heijde scoring method for PsA

The Sharp-van der Heijde score modified for PsA (SvdH; total score range, 0–528) also assesses joints of the hands, wrists, and feet and includes DIP joints 2 to 5 of both hands (Table 1) [3, 4]. Scores for erosion range from 0 to 5 in the hands and 0 to 10 in the feet and reflect erosion size, with 0 defined as no erosion and 3 defined as a large erosion passing the midline of the joint. If there is > 1 erosion per joint, scores can be combined to give a maximum score of 5 per joint in the hands and 10 per joint in the feet (a maximum of 5 at each side of the joint). Joint space narrowing scores vary from 0 to 4 in both the hands and feet, with 0 being normal and 4 being the absence of joint space with evident ankylosis or subluxation [3, 4]. Gross osteolysis and pencil-in-cup change are scored separately and, if present, are assigned the maximum score for erosion and joint space narrowing for the same affected joint [3].

#### Psoriatic Arthritis Ratingen score

The Psoriatic Arthritis Ratingen score (PARS) is the only scoring system that was specifically designed for PsA and may better account for the bone pathology changes (destruction and proliferation) seen in these patients [3, 4, 14]. Destruction is scored from 0 to 5 and measures percentage of joint surface destruction, with a score of 5 reflecting destruction of > 75% of the joint surface (Table 1). Proliferation is scored from 0 to 4 and measures bony outgrowth in millimeters, also accounting for the size of the new growth relative to the original diameter of the bone [3, 4]. The PARS score includes assessment of 30 joints in the hands and 10 joints in the feet, with a potential proliferation score of 0 to 200 and destruction score of 0 to 160, yielding a total score ranging from 0 to 360 [3, 4].

### Comparison of scoring methods

Although all four methods (the modified Steinbrocker scoring method, the modified Sharp score, the SvdH method, and the PARS) are reliable in the assessment of radiographic progression, no direct comparison of their performance in clinical trials has been conducted. The modified Steinbrocker scoring method is generally easier to learn and use but is less sensitive than methods based on the modified Sharp score; this lower level of sensitivity may be one reason the modified Steinbrocker method has not been routinely used in clinical trials [33]. The SvdH method for PsA has been used to evaluate treatment in several studies of biologic agents and has shown good sensitivity and reliability (Table 2) [19–30, 32, 34].

A study by Tillet and colleagues compared the four scoring methods to assess their utility in PsA [33]. Radiographs of hands and feet from 50 patients with PsA were scored at two time points by two readers using each of the four methods. Sensitivity to change was estimated using a standardized response mean and smallest detectable change. The SvdH method was the most reliable and sensitive to change but required a longer time to perform. The Steinbrocker method was the most feasible (i.e., took the least time to perform) but was not as sensitive as the SvdH or modified Sharp score. The authors concluded that the SvdH scoring method for PsA was the best tool for use in randomized controlled trials. Although a case study has shown that scoring methods can detect changes in radiographic damage over time in patients with PsA [35], Tillet and colleagues found that none of the methods currently available provide an adequate level of feasibility and sensitivity for application in large, longitudinal studies [33]. The importance of meeting this need is highlighted in the European League Against Rheumatism (EULAR) research agenda, which seeks to define the optimal use of radiographic scores for monitoring structural damage progression in longitudinal studies of PsA [11].

## Assessment of structural damage progression in clinical research

### Structural damage as an outcome measure in clinical trials

The utility of radiographic assessment of structural damage as an outcome in PsA is illustrated by several phase 3 clinical trials of biologic agents used to treat PsA (Table 2) [15, 17, 19–32, 34, 36]. The first studies of the biologic agents etanercept [15] and adalimumab [17] in PsA used a modified Sharp score for PsA to assess the effect of therapy on structural damage. In the study evaluating etanercept vs placebo, the primary radiographic endpoint was the annualized rate of change in the modified Sharp score for PsA at 6 and 12 months [15]. At 12 months, etanercept led to inhibition of radiographic progression in the hands and wrists compared with worsening in the placebo group (*P* = .0001); annualized changes in the erosion score and joint space narrowing scores were also significantly different in the two groups. In the ADEPT study of adalimumab vs placebo, inhibition of structural damage, as measured by the Sharp method for PsA at week 24, was a primary efficacy endpoint [17]. At week 24, active treatment demonstrated significant inhibition of structural damage progression compared with placebo (*P* < .001). Significant differences were also observed in erosion and joint space narrowing scores.

Since then, other trials of biologics in PsA have used the SvdH scoring system (Table 2). The IMPACT studies were some of the first studies to use the SvdH method to assess structural damage in patients with PsA being treated with a biologic agent (infliximab) [19, 20]. The primary outcome was mean progression of structural damage as demonstrated by the mean changes from baseline in the total SvdH score, with positive changes from baseline indicating progression of structural damage. The studies showed that the SvdH scoring method was appropriate for use in PsA; however, the usefulness of scoring features characteristic of PsA (e.g., hand DIP joints, pencil-in-cup changes, gross osteolysis) was limited, as had been observed previously when using the Sharp method in PsA [15, 17]. This was mainly due to the low progression rate of these features over 6 to 12 months. Importantly, the IMPACT studies showed that biologic treatments were able to inhibit radiographic progression as early as 6 months, and therefore assessment of structural damage in clinical trials can be performed after 6 months as opposed to waiting 1 to 2 years to assess damage. These studies also suggested that for ethical reasons, patients in clinical trials should not be offered placebo for long periods of time but should be allowed to receive active treatment after a 6-month period.

In line with these findings, the GO-REVEAL [21] and GO-VIBRANT [22] studies of golimumab showed that 24 weeks of follow-up are sufficient for radiographic damage as an outcome. In these studies, the change from baseline in the SvdH scoring method for PsA of the hands and feet at week 24 was one of the two coprimary endpoints (Table 2). Scores for PsA-specific radiologic damage (e.g., DIPs, pencil-in-cup, and gross osteolysis deformities) were also included. As seen in the IMPACT studies, patients who originally received placebo and later crossed over to receive active treatment had more structural damage after 1 year than did patients who originally received active treatment, suggesting a benefit associated with earlier treatment.

As new targeted therapies for PsA have been developed, inhibiting radiographic progression has become essential for demonstrating efficacy and disease-modifying activity. For instance, the phase 3 studies PSUMMIT-1 and PSUMMIT-2 of the anti–interleukin (IL) 12/IL-23 antibody ustekinumab assessed changes from baseline in radiographic progression at week 24 using the SvdH method for PsA (Table 2) [25]. Findings from this study showed that inhibiting targets other than tumor necrosis factor (i.e., IL-12 and IL-23) could also lead to improvements in PsA and inhibition of radiographic progression. In the FUTURE studies of secukinumab (an anti–IL-17 inhibitor), inhibition of radiographic progression was a key secondary endpoint [26, 27, 34]. Patients treated with secukinumab had significantly less radiographic progression, defined as the change from baseline in SvdH score at week 24. Inhibition of radiographic progression was sustained up to 2 years for both erosion and joint space narrowing [26, 37]. These studies demonstrated that targeting IL-17A was another therapeutic option for patients with PsA. Similar assessments of radiographic progression were conducted in the SPIRIT-P1 study of ixekizumab, another IL-17 inhibitor [28, 32], and in studies of the Janus (JAK) inhibitor tofacitinib [30] and the T cell modulator abatacept [29] (Table 2).

The impact of methotrexate on radiographic outcomes has also been assessed. The phase 3 randomized study SEAM-PsA, which examined the efficacy of methotrexate and etanercept monotherapies vs methotrexate in combination with etanercept [31, 36] (Table 2), found that concomitant methotrexate did not lead to significant changes in radiographic outcomes, consistent with previous observations [15, 26]. However, in studies of golimumab [21] and infliximab [20], treatment group differences were larger in patients receiving concomitant methotrexate.

### Limitations and considerations in the analysis of radiographic progression in clinical trials

Given that it is based on subjective interpretation of radiographic changes, the assessment of radiographic progression is subject to possible measurement error and variability. In general, the presence of radiographic damage in clinical trials is preferably assessed by 2 or 3 central readers to ensure reliable information [38]. Additionally, a mean change of ≤ 0.5 in total score (vs 0) is preferred when the mean of two readers is used to determine the absence of radiographic progression.

A main challenge in assessing radiographic progression is detecting a treatment effect [39, 40]. Ethical considerations in clinical trials limit the duration of placebo treatment, impacting the ability to detect radiographic progression in this group. Additionally, patients randomized to placebo are allowed to switch to active treatment due to lack of efficacy or to discontinue the study before radiographic progression is assessed or discernable. These low rates of radiographic progression in the control group and incomplete radiographic data may impact the statistical power of these trials to detect a treatment effect [39].

Another important challenge is mitigating the impact of missing radiographic data. Linear extrapolation and interpolation is a widely used approach and requires radiographic data from ≥ 2 time points [23, 27]. Linear mixed-effects models, which account for important cofactors such as previous treatment and baseline values, are another approach that can maximize the statistical power of clinical trials and mitigate the effect of missing data. These methodologies have been used in various studies. For example, in the IMPACT-2 study, missing data were imputed using linear extrapolation or the median of the change in total scores based on all patients within the same methotrexate stratification (i.e., a median of 0) [20]. In the GO-REVEAL study, linear extrapolation was used, and if data were insufficient for linear extrapolation, the median change in total SvdH score was used to replace missing data [21]. However, imputation of median scores is no longer applied. In the FUTURE5 study, radiographic data were analyzed by a linear mixed-effects model that excluded data after escape for patients treated with placebo who received escape therapy at week 16. The model assumed approximately linear progression over time and estimated a difference in rates of progression over 24 weeks to compare treatment groups [41]. However, it is important to note that imputation methodologies may significantly influence interpretation of radiographic outcomes, as seen in the RAPID-PsA phase 3 study of certolizumab pegol, where (incorrect) imputation methodologies resulted in a high degree of progression in all arms [23]. In this study, not imputing missing data for patients with ≤ 1 radiograph or 2 radiographs < 8 weeks apart and linear extrapolation in patients with two radiographs ≥ 8 weeks apart were found to be the most appropriate methods for the primary analysis.

In addition to imputation methodologies, enriching for patients who are at high risk of radiographic progression may increase the power of a study to detect treatment effects [39]. This could be achieved by increasing the number of patients who present with predictive factors for radiographic progression or by indirectly enriching the data through post hoc analyses. Although predictive factors for radiographic progression in PsA are limited, systemic inflammation as indicated by elevated baseline C-reactive protein has been shown to be a strong independent predictor of radiographic progression and may serve as a way of enriching for high-risk patients [42, 43]. Similarly, the existence of radiographic damage is another predictive factor. Patients with damage are more prone to develop more damage, especially in the presence of an elevated C-reactive protein.

Another limitation of clinical trials that assess structural damage in PsA is that they tend to focus on radiographic progression in peripheral joints only. For instance, structural changes associated with enthesitis, including anabolic bone formation, are not generally assessed, and no systematic method of measurement is currently available [12]. Similarly, progression in the axial skeleton is not commonly measured. Different scoring methods for assessment of axial involvement are available [44]; however, they have not been used in large clinical trials of PsA so far. A further limitation is that minimum clinically important differences for radiographic scores have not yet been established.

### Future areas of research

Scoring methods for other imaging techniques (i.e., micro-computed tomography scan, ultrasound, MRI) should be developed, validated, and further tested for their propensity to assess structural damage in PsA [11]. So far, only MRI has been used and is scored using the Psoriatic Arthritis Magnetic Resonance Image score (PsAMRIS) [45, 46]. PsAMRIS is a semiquantitative scoring system that has been developed for PsA by the international MRI in arthritis group of OMERACT (Outcome Measures in Rheumatology) [45, 46] and is the most validated system available for the evaluation of inflammatory and structural changes in the hands of patients with PsA [45]. PsAMRIS has been used in a few small trials in which a significant improvement in inflammatory parameters was demonstrated following treatment with a biologic agent; however, bone damage parameters, such as bone proliferation and erosion, showed little change over time [45]. New developments in MRI approaches, such as dynamic contrast-enhanced MRI and digital automated analysis, may improve MRI techniques [47].

Additionally, further refinement of radiographic scoring methods specific for PsA merits investigation. For example, radiographic scoring methods could be improved by accounting for the progression of new bone growth, which is characteristic of PsA. Another area of development could be the use of artificial intelligence to assess structural damage on radiographs.

## Conclusions

Structural damage in patients with PsA is associated with decreased quality of life and physical function and increased risk of death. Therefore, inhibition of structural damage progression is a key aspect of treatment and a characteristic of disease-modifying therapies in PsA. This has led to radiographic progression becoming an important outcome measure in clinical trials that is needed to identify effective therapies for patients with PsA. As sustained inhibition of radiographic progression becomes an important goal in the management of the disease, further research will be necessary to determine the best way of assessing radiographic progression long term.

## Data Availability

Data sharing is not applicable to this article as no datasets were generated or analyzed during the current study

## Abbreviations

DIP: Distal interphalangeal
ETN: Etanercept
EULAR: European League Against Rheumatism
IL: Interleukin
IXE: Ixekizumab
LD: Loading dose
MRI: Magnetic resonance imaging
SvdH: Sharp-van der Heijde score modified for PsA
MTX: Methotrexate
OMERACT: Outcome Measures in Rheumatology
PARS: Psoriatic arthritis Ratingen score
PBO: Placebo
PsA: Psoriatic arthritis
PsAMRIS: Psoriatic Arthritis Magnetic Resonance Image score
Q2W: Every 2 weeks
Q4W: Every 4 weeks
TOFA: Tofacitinib
